# Maize Endophytic Bacterial Diversity as Affected by Soil Cultivation History

**DOI:** 10.3389/fmicb.2018.00484

**Published:** 2018-03-16

**Authors:** David Correa-Galeote, Eulogio J. Bedmar, Gregorio J. Arone

**Affiliations:** ^1^Department of Soil Microbiology and Symbiotic Systems, Estación Experimental del Zaidín, Agencia Estatal Consejo Superior de Investigaciones Científicas, Granada, Spain; ^2^Department of Agricultural Sciences, National University of Huancavelica, Huancavelica, Peru

**Keywords:** biodiversity, endophytes, maize, Quechua region, 16S rRNA, pyrosequencing, PGPR bacteria

## Abstract

The bacterial endophytic communities residing within roots of maize (*Zea mays* L.) plants cultivated by a sustainable management in soils from the Quechua maize belt (Peruvian Andes) were examined using tags pyrosequencing spanning the V4 and V5 hypervariable regions of the 16S rRNA. Across four replicate libraries, two corresponding to sequences of endophytic bacteria from long time maize-cultivated soils and the other two obtained from fallow soils, 793 bacterial sequences were found that grouped into 188 bacterial operational taxonomic units (OTUs, 97% genetic similarity). The numbers of OTUs in the libraries from the maize-cultivated soils were significantly higher than those found in the libraries from fallow soils. A mean of 30 genera were found in the fallow soil libraries and 47 were in those from the maize-cultivated soils. Both alpha and beta diversity indexes showed clear differences between bacterial endophytic populations from plants with different soil cultivation history and that the soils cultivated for long time requires a higher diversity of endophytes. The number of sequences corresponding to main genera *Sphingomonas, Herbaspirillum, Bradyrhizobium* and *Methylophilus* in the maize-cultivated libraries were statistically more abundant than those from the fallow soils. Sequences of genera *Dyella* and *Sreptococcus* were significantly more abundant in the libraries from the fallow soils. Relative abundance of genera *Burkholderia, candidatus* Glomeribacter, *Staphylococcus, Variovorax, Bacillus* and *Chitinophaga* were similar among libraries. A canonical correspondence analysis of the relative abundance of the main genera showed that the four libraries distributed in two clearly separated groups. Our results suggest that cultivation history is an important driver of endophytic colonization of maize and that after a long time of cultivation of the soil the maize plants need to increase the richness of the bacterial endophytes communities.

## Introduction

Chacras are small (200–10,000 m^2^) plots in the Quechua region of the Peruvian Andes where maize, pea, wheat, potatoes and other vegetables and cereals are cultivated by the native peasants. Commodities that they use for their own consumption and for trade in local and regional markets. Genetic and archeological data indicate that after domestication in Mexico about 8,700 years before the present (cal. BP) (Piperno et al., 2009; Van Heerwaarden et al., 2011; Grobman et al., 2012), maize spread in other Mexican regions and into south of Mexico, reaching the southern Andean highlands by 4,000 before the present (Perry et al., 2006). Since then maize is the staple diet of the Quechua natives who continue growing it as their ancestors did. Mostly without chemical fertilization, no pesticide application and without irrigation and yet chacras maintain a sustainable production for years.

Bacterial endophytes have been defined as microorganisms that could be isolated from surface-sterilized plant tissues and do not visibly harm host plants (Petrini, 1991; Hallmann et al., 1997; Schulz and Boyle, 2006). It is now considered that endophytism is a universal phenomenon (Kobayashi and Palumbo, 2000) and is likely that all plants harbor endophyte bacteria (Rosenblueth and Martínez-Romero, 2006; Ryan et al., 2008; Compant et al., 2010; Dudeja and Giri, 2014). Endophytes have been involved in plant growth promotion, biological control of plant pathogens, isolation of compounds of pharmaceutical or biotechnological interest (reviewed in Schulz, 2006; Weyens et al., 2009; Li et al., 2012; Malfanova et al., 2013; Hardoim et al., 2015; Ma et al., 2016; Vejan et al., 2016; Sharma et al., 2017).

Previous studies have analyzed bacterial taxa associated with maize. Most of the work has been done by using culture-dependent methods (Rai et al., 2007; Rijavec et al., 2007; Pereira et al., 2009, 2011; Ikeda et al., 2013; Celador-Lera et al., 2016; Menéndez et al., 2016; Sandhya et al., 2017) or assessed bacterial diversity independently of culture approaches (Schmalenberger and Tebbe, 2003; Herschkovitz et al., 2005a,b; Pereira et al., 2011; Correa-Galeote et al., 2016; Liu et al., 2017). The high-throughput pyrosequencing technology introduced by 454 Life Science (Margulies et al., 2005; Rothberg and Leamon, 2008) has been used to assess diversity in cultivar-specific bacterial endophyte communities in potato roots (Manter et al., 2010), leaf vegetables (Jackson et al., 2013), the spermosphere and phyllosphere of spinach (López-Velasco et al., 2013), tomato leaves (Romero et al., 2014), grapevine leaves and stems (Yousaf et al., 2014) and roots and shoots of cucumber (Eevers et al., 2016).

Most of the works has been done toward understand the effect of an engineered varieties of maize in the bacterial diversity in maize rhizosphere (Schmalenberger and Tebbe, 2003; Ikeda et al., 2013; Liu et al., 2017) or to analyse the changes in the rhizosphere of maize after the application of an inoculant (Herschkovitz et al., 2005a,b; Sanguin et al., 2006a,b; Alves et al., 2015). In recent years, sustainability agriculture methods have also been considered as great potential source of new information and perspective in agriculture and food systems (Wezel et al., 2011). Bacterial diversity is central to ecosystem sustainability and soil biological function, for which the role of roots is especially important (Sanguin et al., 2006b). However, the characterization of bacterial endophyte community of agroecology systems as is the Quechua practices has been poorly analyzed.

In this work we have analyzed the bacterial endophytes that inhabit the roots of maize plants grown under a different management history in the Quechua region by pyrosequencing the V4 and V5 hypervariable regions of the 16S rRNA gene. Our hypothesis was that the soil cultivation history plays a pivotal role of structuring the endophytic bacterial communities of maize plants.

## Materials and methods

### Site description and root sampling

Maize (*Zea mays* L.) plants were grown at 4 chacras located inside the same farm field (Figure S1A) near Allpas (12° 50′ 27″ S, 74° 34′ 14″ W, at 3,537 m above sea level), a village in the province of Acobamba (Huancavelica, Peru), following the traditional agricultural practices of the Quechua natives. The lateral roots (~2 mm diameter, 2–3 cm long) of the maize plants (morphotype Qarway) were harvested 120 days after sowing. At the sampling time two of the four chacras had been cultivated with maize for at least 5 years (MC soil) and the other two were under fallow conditions before the maize sowing (F soil) for at least 5 years (Figure S1B). For each chacra, roots were sampled from plants grown at three different sites (four plants per site), pooled together, washed with sterile tap water to remove attached soil and stored at −20°C until further processing. Physicochemical analyses of the four chacras indicated they have identical soil characteristics with a sandy loam texture (64.0% sand, 30.0% silt, 6% clay), pH 5.74, 1.6% organic C and 0.11% total N (Table S1).

### Surface sterilization of maize roots and isolation of endophytes

Unfrozen roots were surface-sterilized as indicated by Liu et al. (2017). Essentially, roots were immersed in 70% ethanol for 3 min, washed with fresh sodium hypochlorite solution (2.5% available Cl^−^) for 5 min, rinsed with 70% ethanol for 30 s and finally washed thoroughly with sterile distilled water. To confirm that the sterilization process was successful, small pieces of roots were cut and placed on Petri dishes containing yeast extract-mannitol (YEM) medium (Vincent, 1970). The plates were examined for bacterial growth after incubation at 30°C for 12 days. Maize roots that were not contaminated as detected by culture-dependent sterility test were used for further experiments.

### Extraction of DNA from maize roots

DNA was extracted from 250 mg of unfrozen tissue as previously indicated (Correa-Galeote et al., 2013). Essentially, after thoroughly cutting with an sterile scalpel, samples were homogenized in 1 ml of extraction buffer containing 100 mM Tris (pH 8.0), 100 mM EDTA, 100 mM NaCl, 1% (w/v) polyvinylpyrrolidone and 2% (w/v) sodium dodecyl sulfate using a 2-ml mini-bead-beater tube containing 0.5 and 0.1 g of 106-μm- and 2-mm-diameter glass beads, respectively, for 60 s at 27 Hz. Cell debris was eliminated by centrifugation (14,000 rpm for 5 min at 4°C). Proteins were removed by treatment with 5 M sodium acetate. After treatment for 12 h with ice-cold isopropanol, nucleic acids were precipitated by centrifugation (14,000 rpm for 30 min at 4°C), washed with 70% ice-cold ethanol, recentrifruged (14,000 rpm for 15 min at 4°C) and air-dried for 30 min. Finally, DNA was purified using GeneClean columns (Qiagen). Quality and size of DNA were checked by electrophoresis on 1% agarose and quantified by spectrophotometry at 260 nm using a Nanodrop spectrophotometer (NanoDrop ND1000).

### Amplification and pyrosequencing of DNA from maize roots

Polymerase chain reaction (PCR) amplification of the hypervariable V4-V5 regions of the 16S rRNA gene was performed over each individual DNA extraction from roots of maize plants grown in F and MC soils using universal primers U519F and U926R (Baker et al., 2003) joined to a multiplex identifier sequence (Binladen et al., 2007; Parameswaran et al., 2007). For each sample, amplicons were generated in several replicate PCRs using mixtures (25 μl) that contained 25 pmol of each primer, 1.8 mM MgCl_2_, 0.2 mM dNTPs, 1 × the corresponding *Taq* buffer, 1 U of *Taq* Master (5 Prime, USA) and 10 ng of the DNA template. The PCR program consisted of an initial denaturation step at 94°C for 4 min, 25 cycles of denaturation at 94°C for 15 s, primer annealing at 55°C for 45 s and extension at 72°C for 1 min, followed by a final step of heating at 72°C for 10 min. Amplicons of the same treatment were pooled together to reduce per-PCR variability and purified using the ultracentrifugal filters Ultracel-100 K membranes (Amicon) according to the manufacturer's instructions. After quantification by Nanodrop ND1000 and visualization of the DNA by agarose electrophoresis, the samples were combined in equimolar amounts and pyrosequenced in a Roche Genome Sequencer FLX system using 454 Titanium chemistry at LifeSequencing S.L. (Valencia, Spain).

### Taxonomic assignment of sequence reads and diversity indexes

Raw sequences were processed through the Ribosomal Database Project (RDP) pyrosequencing pipeline (http://pyro.cme.msu.edu) release 11 (Cole et al., 2014). Sequences were trimmed for primers, filtered and assigned to four libraries (F1G, F2G, MC1G and MC2G) according to their tags. Sequences shorter than 150 base pair, with quality scores <20 or containing any unresolved nucleotides were removed from the dataset. Chimeras were identified using the Uchime tool from FunGene database (Edgar et al., 2011) and removed from the dataset. Sequences were aligned using the SILVA-based bacterial reference alignment in the MOTHUR program (Schloss and Westcott, 2011). Aligned sequences were clustered into operational taxonomic units (OTUs) defined at 97% similarity cutoff using MOTHUR and their relative abundances calculated. The number of sequences in each OTU was employed to calculate the Good's coverage index, which is considered a relative measure of how well the sequences obtained represent the entire populations (Hughes and Bohannan, 2004). Taxonomic assignation of the sequences was performed using Geneious (Biomatters). Shannon (H′) and Simpson (S′) diversity indexes and Jaccard indexes (*J*_*class*_ and *J*_*abund*_) were used to analyze the alpha- and beta-diversity, respectively (Chao et al., 2005).

### Statistical analyses

Relative abundances of the main genera and values of the diversity index were compared using the Student *t*-test in the XLSTAT software (Addinsoft). Multivariate techniques were used to analyze the relative abundance of endophytes using PC-ORD (McCune et al., 2002). A canonical correspondence analysis (CCA) was built to study differences in composition of dominant endophyte genera in roots from plants grown in F and MC soils.

### Accession numbers

Pyrosequencing reads are deposited in GenBank under accession numbers KT764133 to KT764925.

## Results

A total of 38,443 sequences were obtained from the four 16S rDNA samples sent to pyrosequencing, of which 11,278 were retained after filtering and removing chimeras. The mean number of total retained sequences per library was 2,819, ranging from 1,770 to 3,718. Average length of retained sequences was 374 ± 5 base pair (mean ± SD). Using the MOTHUR program all the sequences aligned correctly in the expected position of the 16S rDNA sequence of *Escherichia coli* and were grouped at 97% similarity in 244 distinct OTUs. A representative sequence from each OTU was sent to NCBI for identification, after which 10,485 sequences were removed as they were identified as Streptophyta-related sequences. The remaining 793 sequences grouped into 188 bacterial OTUs, of which 17 were supported by 10 or more reads and 91 corresponded to singletons (Table S2). Values of the Good's coverage index were higher than 68% for all the samples. The number of OTUs in libraries F1G and F2G were 48 and 53, respectively, significantly lower than those of 88 and 112 found in libraries MC1G and MC2G (Table 1). The Shannon index for OTUs in F1G and F2G showed similar values, 3.32 and 3.62, respectively, that were statistically lower than those of 4.04 and 4.02 for OTUs in MC1G and MC2G, respectively. On the other hand, values of the Simpson index for the OTUS for the four libraries varied between 0.23 and 0.32, and no significant differences were found among them (Table 1). The Jaccard index for *J*_*class*_ and *J*_*abund*_ also showed that the degree of similarity between libraries MC1G and MC2G was higher than that between F1G and F2G (Table 2). The number of shared genera between pair to pair libraries is shown in Table 2.

**Table 1:** Number of OTUs, values of Good's coverage index and Shannon and Simpson biodiversity index of bacterial endophytes from roots of maize plants grown in fallow (F1 and F2) and maize-cultivated (MC1 and MC2) soils.

**Diversity index**	**F1G**	**F2G**	**MC1G**	**MC2G**	***p*-value of *t*-test**
Number of OTUs	48b	53b	88a	112a	0.06
Good's coverage	77.05	68.47	75.00	81.74	n.a.
Shannon	3.32b	3.62b	4.04a	4.02a	0.06
Simpson	0.052a	0.031a	0.023a	0.030a	0.34

*Values in the same row followed by different letters are statistically different according to the Student's t-test (α ≤ 0.1). n.a., not applicable*.

**Table 2:** Number of shared genera between clone libraries, and Jaccard similarity index using genera presence/absence (*J*_*class*_) and relative abundances (*J*_*abund*_) of the bacterial endophyte communities from roots of maize plants grown in fallow (F1 and F2) and maize-cultivated (MC1 and MC2) soils.

	**Number of shared genera**	***J_*class*_***	***J_*abund*_***
F1G-F2G	14	0.30	0.62
F1G-MC1G	18	0.31	0.61
F1G-MC2G	18	0.31	0.66
F2G-MC1G	14	0.23	0.53
F2G-MC2G	19	0.32	0.66
MC1G-MC2G	32	0.70	0.77

**Figure 1:**
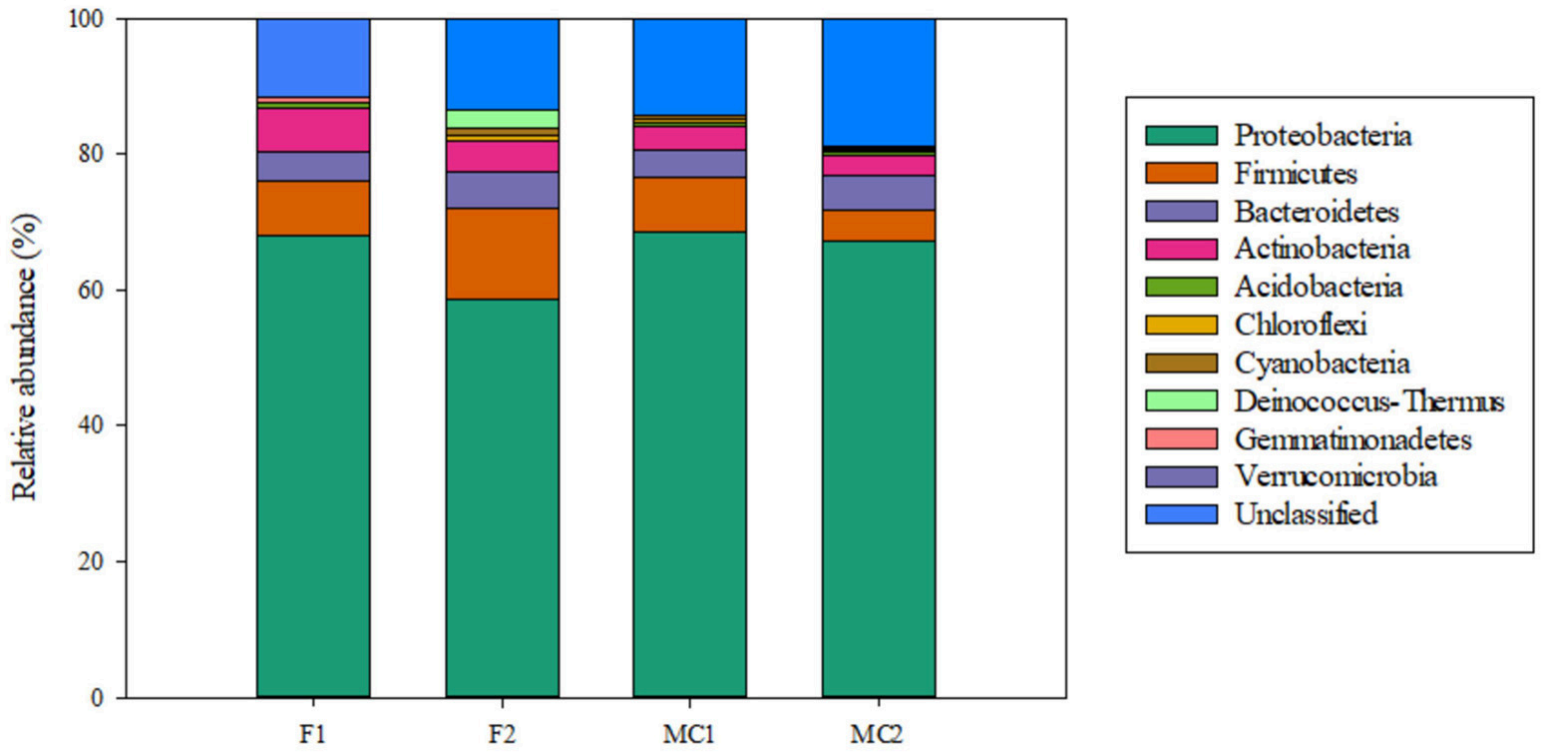
Relative abundance of bacterial endophytes from roots of maize plants grown in fallow (F1 and F2) and maize-cultivated (MC1 and MC2) soils.

Unclassified sequences were 14 (11.48%) and 15 (13.51%) for libraries F1G and F2G, respectively, and 28 (13.73%) and 64 (17.98%) for MC1G and MC2G, respectively (Table 3, Figure 1). The remaining sequences distributed into 6 and 7 phyla for F1G and F2G, respectively, and 7 and 8 for MC1G and MC2G, respectively (Table 3). Phyla Proteobacteria, Firmicutes, Bacteroidetes, Actinobacteria, Acidobacteria, Chloroflexi and Cyanobacteria (in decreasing abundance) were found in each one of the four libraries, Deinococcus-Thermus and Gemmatimonadetes were detected only in libraries FG and Verrucomicrobia was found exclusively in endophytes from libraries MCG (Figure 1). The number of classes, orders, families and genera are also shown in Table 3.

**Table 3:** Number of taxa and distribution of sequences (%) of bacterial endophytes in roots of maize plants grown in fallow (F1 and F2) and maize-cultivated (MC1 and MC2) soils.

	**Fallow soil**				**Maize-cultivated soil**			
	**F1**		**F2**		**MC1**		**MC2**	
	**Number of taxa**	**Number of sequences (%)**	**Number of taxa**	**Number of sequences (%)**	**Number of taxa**	**Number of sequences (%)**	**Number of taxa**	**Number of sequences (%)**
Phylum	6	108 (88.52)	7	96 (86.49)	7	176 (86.27)	8	292 (82.02)
Class	8	107 (87.70)	10	92 (82.88)	8	168 (82.35)	9	285 (80.06)
Order	15	105 (86.07)	15	89 (80.18)	18	154 (75.49)	17	274 (76.97)
Family	24	99 (81.15)	21	79 (71.17)	33	151 (74.02)	33	264 (74.16)
Genus	30	99 (81.15)	30	81 (72.97)	46	147 (72.06)	48	260 (73.03)
Unclassified sequences		14 (11.48)		15 (13.51)		28 (13.73)		64 (17.98)

**Figure 2:**
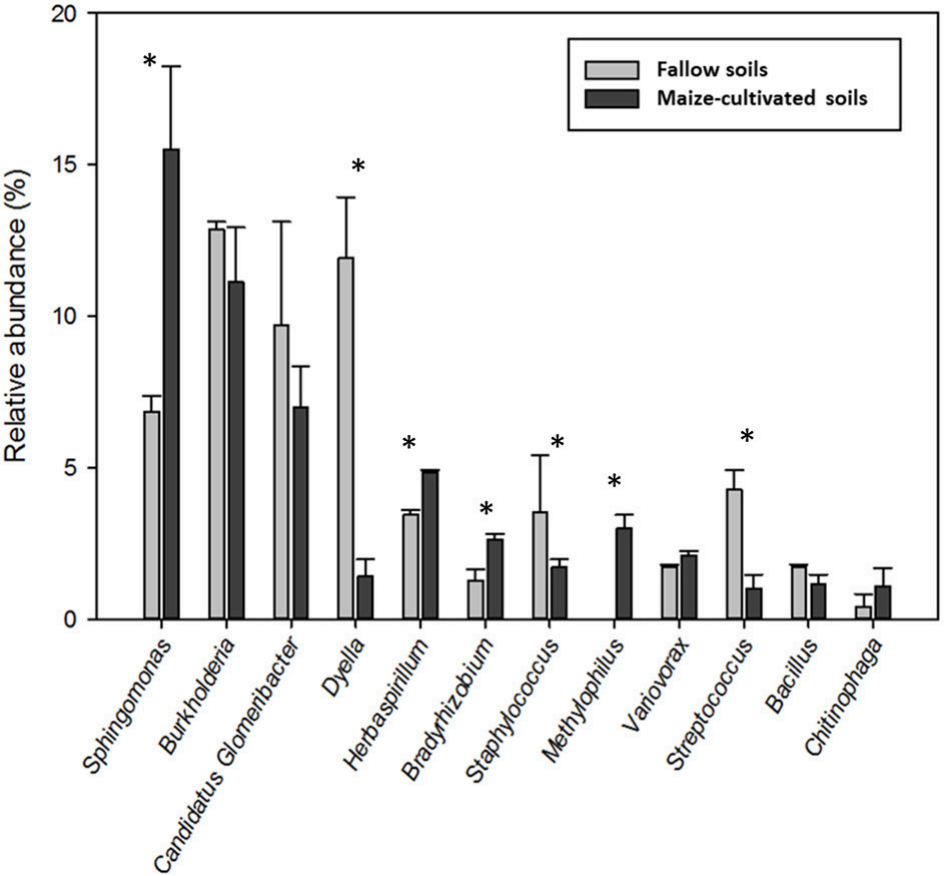
Relative abundance of the 12 main genera found in roots of maize plants grown in fallow and maize-cultivated soils. ^*^Indicates statistically significant differences according to the Student's *t*-test (α ≤ 0.1).

The total 188 OTUs distributed into 82 different genera (Table S2), 12 of which showed a relative abundance higher than 1% and represented the 55% of the total endophytes. Altogether, these genera were (in decreasing order of abundance) *Sphingomonas, Burkholderia, Candidatus* Glomeribacter, *Dyella, Herbaspirillum, Bradyrhizobium, Staphylococcus, Methylophilus, Variovorax, Streptococcus, Bacillus* and *Chitinophaga* (Table S2). The number of sequences corresponding to genera *Sphingomonas, Herbaspirillum, Bradyrhizobium* and *Methylophilus* in libraries MCG were statistically (α ≤ 0.1) more abundant than those in the F libraries, and sequences of genera *Dyella* and *Sreptococcus* were significantly more abundant in the F libraries (Figure 2). Relative abundance of genera *Burkholderia, candidatus* Glomeribacter, *Staphylococcus, Variovorax, Bacillus* and *Chitinophaga* were similar among libraries (Figure 2).

**Figure 3:**
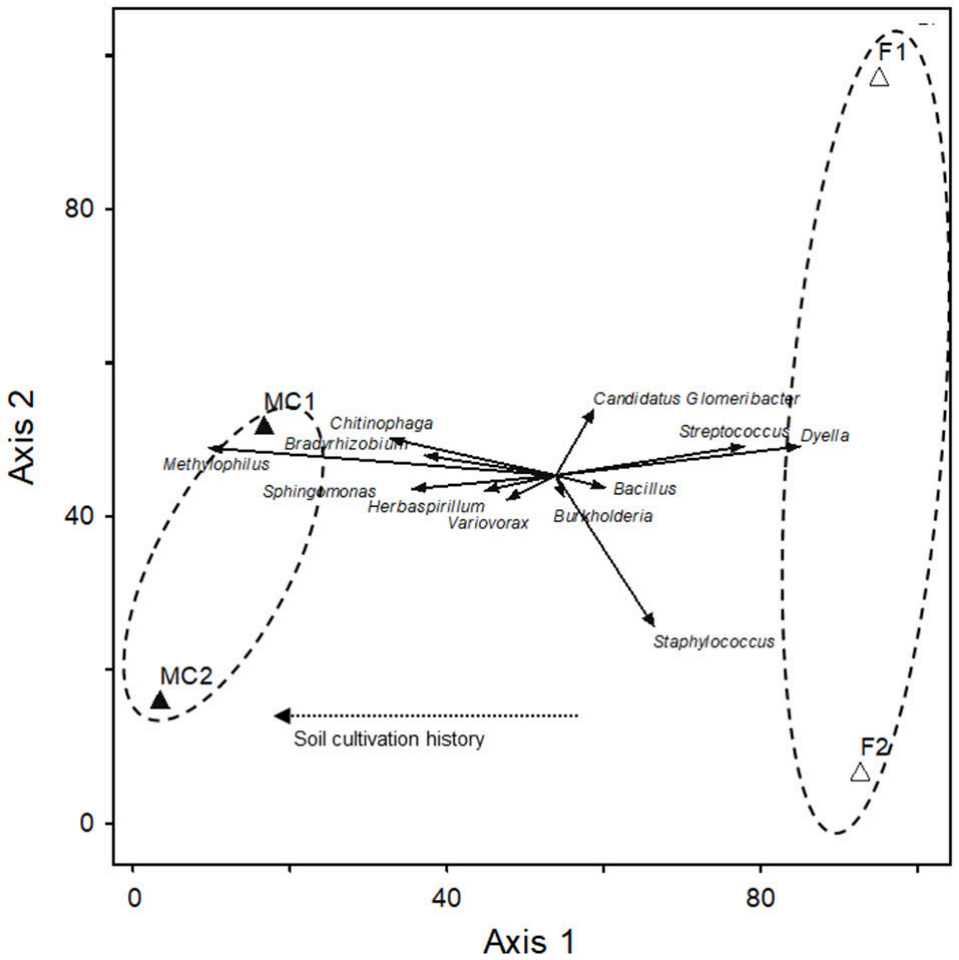
Canonical correspondence analysis (CCA) of the 12 main genera found in roots of maize plants grown in fallow and maize-cultivated soils. Solid arrows represent vector scores for the different genera. Open and closed triangles represent the axes 1 and 2 scores for the main genera found in fallow (F1 and F2) and maize-cultivated (MC1 and MC2) soils, respectively. The dashed arrow represents the biplot vector for cultivation history of the soil.

A CCA sample ordination based on the relative abundance of the 12 main genera mentioned above showed that they distributed in two clearly separated groups (Figure 3). The two CCA axes explained 93% of the total variance and revealed that cultivation history of the soil was responsible for the grouping of the libraries along the axis 1 (canonical coefficient 1.10).

## Discussion

One of the most successful soil management techniques in agricultural land is the use of fallow periods (Costa et al., 2015), this work is a first approach to understand the role of the soil cultivation history in the bacterial diversity of the endophytic bacteria of maize plants cultivated under sustainably practices. Using 454 next generation sequencing we assessed the composition and abundance of endophytic communities inside roots of amilaceous maize plants grown under fallow and maize-cultivated conditions in Andean chacras. Pyrosequencing revealed an unprecedented number of bacterial endophytes as compared with those of the genera found in previous studies based on culture-dependent and culture-independent methods (McInroy and Kloepper, 1995; Chelius and Triplett, 2001; Rai et al., 2007; Pereira et al., 2011; Ikeda et al., 2013; Sandhya et al., 2017). Altogether, a 15.26% of the total sequences found inside roots corresponded to unclassified bacteria, which indicates the presence of hitherto uncultured bacterial groups. Nevertheless, despite the resolving power of pyrosequencing to detect phylogenetic groups, genera *Pantoea, Klebsiella* and *Erwinia* found by other authors (Pereira et al., 2011; Montañez et al., 2012; Ikeda et al., 2013; Liu et al., 2017) after sequencing of the 16S rRNA gene of endophytes isolated from roots of different maize genotypes were not detected in our libraries. This could be due to qualitative differences in endophytic colonization (Ikeda et al., 2013).

A variety of bacteria have been reported to be endophytic, among them mostly Proteobacteria, but also Firmicutes, Actinobacteria and Bacteroidetes (reviewed in Rosenblueth and Martínez-Romero, 2006; Bulgarelli et al., 2013; Malfanova et al., 2013; Hardoim et al., 2015; Liu et al., 2017). In our study, regardless of the cultivation history of the soil, members of phylum Proteobacteria were the most abundant followed by those of Firmicutes, Bacteroidetes and Actinobacteria.

Number of OTUs and Shannon index values were statistically higher for libraries MC1G and MC2G than those for libraries FG1 and FG2. However, although the *J*_*class*_ and *J*_*abund*_ indexes were also higher for the MC communities, the bacterial endophytic communities within the plant roots from MC soils were more similar. Eleven out the 12 main genera were present in both MC and F soils and four of them (*Sphingomonas, Herbaspirillum, Bradyrhizobium* and *Methylophilus*) had increased relative abundance in the MC soils in comparison with that in the F soils. These results, together with those of the clone libraries diversity, indicate that plant cultivation history could have a pivotal role responsible for selection of roots endophytes from rhizospheric bacterial reservoirs. Also, these results could indicated that the maize plant growth in soils cultivated for long time requires a higher diversity of endophytes than the plants grown in a soil under a fallow time due that the natural resources of the soil are depleted after 5 years of cultivation. For example, excessive cultivation can wreck the structure of soil by reducing the capacity of holding enough moisture for growing plants (FAO, 1994) and also has been demonstrate that after 3 years of cultivation organic C, N and P declined about a 25% (Bowman et al., 1990). According to Wood et al. (2017), the fallow period is a key determinant of vegetation and soil dynamics as this period renew soil fertility, biomass and biodiversity. Therefore, after a long-time cultivation the maize plants needs a higher presence of endophytes to minimize the depletion of the soil resources.

Bacterial endophytes have been shown to modulate plant growth and development through N_2_ fixation, solubilization of insoluble phosphorus, production of siderophores, production of phytohormones, lowering of ethylene concentration, production of antibiotics and antifungal metabolites and inducing systemic resistance (Somers et al., 2004; Hardoim et al., 2008, 2015; Ahemad and Kibret, 2014; Vejan et al., 2016). Some genera in this study have been shown to be diazotrophic bacteria (*Sphingomonas, Burkholderia, candidatus* Glomeribacter, *Herbaspirillum, Bradyrhizobium* and *Bacillus*), others solubilize inorganic phosphorus (*Sphingomonas, Burkholderia, Herbaspirillum, Bradyrhizobium, Staphylococcus, Methylophilus, Variovorax, Streptococcus, Bacillus* and *Chitinophaga*), are siderophore (*Sphingomonas, Burkholderia, Herbaspirillum, Bradyrhizobium, Staphylococcus, Methylophilus, Variovorax, Streptococcus, Bacillus* and *Chitinophaga*) or indole acetic acid (*Sphingomonas, Burkholderia, Dyella, Herbaspirillum, Bradyrhizobium, Staphylococcus, Methylophilus, Variovorax, Bacillus* and *Chitinophaga*) producers, have 1-aminocyclopropane-1-carboxylate (ACC) deaminase activity (*Sphingomonas, Burkholderia, Dyella, Herbaspirillum, Bradyrhizobium, Staphylococcus, Variovorax* and *Bacillus*) and are involved in biocontrol activity (*Sphingomonas, Burkholderia, candidatus* Glomeribacter, *Herbaspirillum, Methylophilus, Variovorax, Bacillus* and *Chitinophaga*; see Table S3).

To our knowledge, 6 of the 12 main genera in this study (*Bradyrhizobium, Variovorax, Chitinophaga, candidatus* Glomeribacter, *Dyella* and *Streptococcus*) have not been reported as endophytes of amilaceous maize. It should be noted, that the bacteria reported as maize endophytes for the first time could present biotechnological implications as is the case of the formulation of new microbial inoculants. The presence of *Streptococcus, Dyella* and *Staphylococcus* is intriguing as they are well-known human pathogens; these three genera have been detected in maize seeds, roots of blackberry, grapevine shoots, apple and orange fresh fruits (Liu et al., 2013; Phukon et al., 2013; Pinto et al., 2014; Yousaf et al., 2014; Contreras et al., 2016) and they were reported as the dominant endophytes of legumes (Boine et al., 2008; Becerra-Castro et al., 2011). Moreover, recent works suggest that pathogenic bacteria are common inhabitants of the interior of plants (Szilagyi-Zecchin et al., 2014; Blain et al., 2017; Sandhya et al., 2017).

There is to note, out of the 12 main endophytic genera here described, the genera *Candidatus* Glomeribacter, *Dyella, Herbaspirillum* and *Streptococcus* were not found in a previous work (Correa-Galeote et al., 2016) that describe the rizhospherics communities of these chacras and therefore the mechanisms of how these bacteria arrive to the interior of the maize roots is still unclear.

The plant host genotype (Ding et al., 2013), soil type (Rasche et al., 2006; Bulgarelli et al., 2013) and environmental soil conditions (Lundberg et al., 2012) among other factors shape bacterial community composition. Because seeds of amilaceous maize used for planting were the same and the environmental conditions, including soil type, soil psychochemical properties and irrigation, were very much alike for the four chacras used in this study, our results suggest that soil cultivation history could be a main factor controlling colonization of the internal root tissues of the plants.

Taken together our results lend support to the suggestion that cultivation history is an important driver of endophytic colonization of maize and that after a long time of cultivation of the soil the maize plants there grown need to increase the richness of the bacterial endophytes communities. Also, these results point to the importance of the fallow period of the traditional and sustainable Quechua agriculture methods in the maintenance of the soil fertility of Peruvian soils.

As a caveat, since richness of bacteria colonizing maize roots was based on pyrosequencing, it is not known whether the detection of bacteria based on DNA signature alone represent active microbes that are interacting with the host plant. Further experiments should be made in order to isolate the main endophytes described in this work and also analyze their role in the development of the maize host plant cultivated under a sustainable method as is the traditional Quechua agriculture.

## Author contributions

DC-G made substantial contributions to the conception and design of the work, acquisition, analysis and interpretation of data, drafting the work and revising it critically for important intellectual content, final approval of the version to be published, agreement to be accountable for all aspects of the work. EB made substantial contributions to the conception or design of the work, interpretation of data, revising it critically for important intellectual content, final approval of the version to be published, agreement to be accountable for all aspects of the work. GA made substantial contributions to the conception or design of the work, revising it critically for important intellectual content, final approval of the version to be published, agreement to be accountable for all aspects of the work.

### Conflict of interest statement

The authors declare that the research was conducted in the absence of any commercial or financial relationships that could be construed as a potential conflict of interest.
