# Supplementary material for: Maize Endophytic Bacterial Diversity as Affected by Soil Cultivation History
Source: Front Microbiol. 2018 Mar 16;9:484. doi: 10.3389/fmicb.2018.00484 (PMC5890191; doi:10.3389/fmicb.2018.00484)
Supplement: Supplementary file 1 [file Table1.docx]

Table S1. pH, total organic carbon (TOC) and total nitrogen (NT) of fallow (F1 and F2) and maize-cultivated (MC1 and MC2) soils. Values in the same row followed by different letters are statistically different according to the ANOVA test (α ≤ 0.05).

|  | F1 | F2 | MC1 | MC2 | *p* value of AVONA test |
| --- | --- | --- | --- | --- | --- |
| pH | 5.72 ± 0.03 a | 5.81 ± 0.04 a | 5.73 ± 0.02 a | 5.69 ± 0.12 a | 0.635 |
| TOC (%) | 1.58 ± 0.055 a | 1.62 ± 0.01 a | 1.58 ± 0.09 a | 1.62 ± 0.07 a | 0.957 |
| TN (%) | 0.11 ± 0.01 a | 0.12 ± 0.01 a | 0.11 ± 0.01 a | 0.12 ± 0.01 a | 0.536 |
