# Supplementary material for: Maize Endophytic Bacterial Diversity as Affected by Soil Cultivation History
Source: Front Microbiol. 2018 Mar 16;9:484. doi: 10.3389/fmicb.2018.00484 (PMC5890191; doi:10.3389/fmicb.2018.00484)
Supplement: Supplementary file 2 [file Table2.docx]

Table S2. Total number of OTUs and their taxonomic classification, and number of bacterial sequences of each OTU found in roots of maize plants grown in fallow (F1 and F2) and maize-cultivated (MC1 and MC2) soils.

| OTU | Phylum | Class | Order | Family | Genus | Number of bacterial sequences | | | |
| --- | --- | --- | --- | --- | --- | --- | --- | --- | --- |
|  |  |  |  |  |  | F1 | F2 | MC1 | MC2 |
| Otu1 | Proteobacteria | Betaproteobacteria | Burkholderiales | Burkholderiaceae | *Burkholderia* | 13 | 11 | 10 | 25 |
| Otu2 | Proteobacteria | Alphaproteobacteria | Sphingomonadales | Sphingomonadaceae | *Sphingomonas* | 1 | 5 | 9 | 24 |
| Otu3 | Firmicutes | Bacilli | Lactobacillales | Streptococcaceae | *Streptococcus* | 6 | 4 | 3 | 2 |
| Otu4 | Proteobacteria | Alphaproteobacteria | Caulobacterales | Caulobacteraceae | *Caulobacter* | 1 | 2 | 1 | 1 |
| Otu5 | Firmicutes | Bacilli | Bacillales |  |  | 2 | 6 | 3 | 7 |
| Otu6 | Unclassified | | | | | 0 | 1 | 1 | 1 |
| Otu7 | Proteobacteria | Alphaproteobacteria | Sphingomonadales | Sphingomonadaceae | *Sphingomonas* | 0 | 0 | 1 | 0 |
| Otu8 | Proteobacteria | Betaproteobacteria | Burkholderiales | Oxalobacteraceae | *Herbaspirillum* | 4 | 4 | 10 | 17 |
| Otu9 | Proteobacteria | Betaproteobacteria | Burkholderiales | Burkholderiaceae | *Burkholderia* | 3 | 3 | 9 | 21 |
| Otu10 | Proteobacteria | Betaproteobacteria |  |  |  | 1 | 2 | 6 | 5 |
| Otu11 | Unclassified | | | | | 1 | 2 | 3 | 4 |
| Otu11 | Unclassified | | | | | 17 | 11 | 4 | 3 |
| Otu13 | Proteobacteria | Betaproteobacteria | Burkholderiales | Burkholderiaceae | *Candidatus*Glomeribacter | 15 | 6 | 2 | 3 |
| Otu14 | Proteobacteria | Betaproteobacteria | Burkholderiales | Burkholderiaceae | *Candidatus*Glomeribacter | 1 | 1 | 15 | 17 |
| Otu15 | Firmicutes | Bacilli | Bacillales | Bacillaceae | *Bacillus* | 2 | 2 | 3 | 3 |
| Otu16 | Bacteroidetes | Sphingobacteriia | Sphingobacteriales | Sphingobacteriaceae | *Mucilaginibacter* | 1 | 1 | 1 | 4 |
| Otu17 | Bacteroidetes | Sphingobacteriia | Sphingobacteriales | Chitinophagaceae | *Chitinophaga* | 1 | 0 | 1 | 6 |
| Otu18 | Unclassified | | | | | 4 | 1 | 3 | 11 |
| Otu19 | Proteobacteria | Alphaproteobacteria | Rhizobiales | Bradyrhizobiaceae | *Bradyrhizobium* | 2 | 1 | 5 | 10 |
| Otu20 | Actinobacteria | Actinobacteria | Actinomycetales |  |  | 2 | 1 | 0 | 3 |
| Otu21 | Unclassified | | | | | 0 | 0 | 2 | 1 |
| Otu22 | Proteobacteria | Betaproteobacteria | Burkholderiales | Comamonadaceae | *Pelomonas* | 0 | 0 | 2 | 1 |
| Otu23 | Bacteroidetes | Flavobacteriia |  |  |  | 1 | 0 | 1 | 0 |
| Otu24 | Proteobacteria | Gammaproteobacteria | Pseudomonadales | Moraxellaceae | *Acinetobacter* | 1 | 0 | 1 | 0 |
| Otu25 | Unclassified | | | | | 2 | 0 | 0 | 1 |
| Otu26 | Proteobacteria | Alphaproteobacteria | Rhizobiales | Rhizobiaceae | *Sinorhizobium* | 1 | 0 | 0 | 2 |
| Otu27 | Proteobacteria | Gammaproteobacteria | Oceanospirillales | Halomonadaceae | *Chromohalobacter* | 2 | 1 | 0 | 0 |
| Otu28 | Proteobacteria | Alphaproteobacteria | Sphingomonadales | Sphingomonadaceae | *Sphingomonas* | 8 | 2 | 9 | 32 |
| Otu29 | Proteobacteria | Alphaproteobacteria | Rhizobiales | Methylocystaceae | *Pleomorphomonas* | 0 | 0 | 2 | 1 |
| Otu30 | Proteobacteria | Betaproteobacteria | Burkholderiales | Oxalobacteraceae | *Massilia* | 0 | 0 | 2 | 2 |
| Otu31 | Proteobacteria | Betaproteobacteria | Burkholderiales | Oxalobacteraceae | *Duganella* | 0 | 1 | 0 | 6 |
| Otu32 | Bacteroidetes | Sphingobacteriia | Sphingobacteriales | Sphingobacteriaceae | *Pedobacter* | 1 | 0 | 1 | 4 |
| Otu33 | Unclassified | | | | | 0 | 0 | 1 | 0 |
| Otu34 | Proteobacteria | Alphaproteobacteria | Rhizobiales | Rhizobiaceae | *Rhizobium* | 0 | 3 | 0 | 4 |
| Otu35 | Firmicutes | Clostridia | Clostridiales | Clostridiaceae | *Clostridium* | 0 | 0 | 1 | 1 |
| Otu36 | Unclassified | | | | | 0 | 0 | 1 | 0 |
| Otu37 | Unclassified | | | | | 1 | 3 | 0 | 2 |
| Otu38 | Firmicutes | Bacilli | Bacillales | BacillalesFamily XI | *Gemella* | 0 | 1 | 0 | 1 |
| Otu39 | Proteobacteria | Betaproteobacteria | Rhodocyclales | Rhodocyclaceae | *Dechloromonas* | 0 | 0 | 2 | 1 |
| Otu40 | Proteobacteria | Betaproteobacteria | Burkholderiales | Comamonadaceae | *Variovorax* | 2 | 2 | 4 | 8 |
| Otu41 | Proteobacteria | Alphaproteobacteria | Sphingomonadales | Sphingomonadaceae | *Sphingomonas* | 0 | 0 | 0 | 6 |
| Otu42 | Proteobacteria |  |  |  |  | 0 | 1 | 0 | 1 |
| Otu43 | Actinobacteria | Actinobacteria | Actinomycetales | Actinomycetaceae | *Actinomyces* | 1 | 0 | 1 | 0 |
| Otu44 | Proteobacteria | Gammaproteobacteria | Xanthomonadales | Xanthomonadaceae | *Stenotrophomonas* | 0 | 1 | 0 | 5 |
| Otu45 | Actinobacteria | Actinobacteria | Actinomycetales | Nocardioidaceae | *Nocardioides* | 0 | 2 | 1 | 3 |
| Otu46 | Proteobacteria | Gammaproteobacteria | Pasteurellales | Pasteurellaceae | *Haemophilus* | 0 | 0 | 2 | 0 |
| Otu47 | Proteobacteria | Gammaproteobacteria | Xanthomonadales | Xanthomonadaceae | *Lysobacter* | 0 | 0 | 2 | 3 |
| Otu48 | Firmicutes | Bacilli | Bacillales | Paenibacillaceae | *Paenibacillus* | 0 | 0 | 1 | 0 |
| Otu49 | Proteobacteria | Gammaproteobacteria |  |  |  | 0 | 0 | 2 | 0 |
| Otu50 | Proteobacteria | Alphaproteobacteria | Rhizobiales | Hyphomicrobiaceae | *Devosia* | 0 | 0 | 1 | 4 |
| Otu51 | Proteobacteria | Alphaproteobacteria | Rhizobiales | Bradyrhizobiaceae | *Bosea* | 0 | 0 | 2 | 0 |
| Otu52 | Unclassified | | | | | 0 | 0 | 0 | 1 |
| Otu53 | Proteobacteria | Betaproteobacteria | Burkholderiales | Oxalobacteraceae | *Collimonas* | 0 | 0 | 0 | 3 |
| Otu54 | Unclassified | | | | | 0 | 0 | 0 | 2 |
| Otu55 | Proteobacteria | Betaproteobacteria | Burkholderiales | Burkholderiaceae | *Ralstonia* | 0 | 0 | 2 | 0 |
| Otu56 | Cyanobacteria |  |  |  |  | 0 | 0 | 1 | 1 |
| Otu57 | Unclassified | | | | | 0 | 0 | 0 | 1 |
| Otu58 | Unclassified | | | | | 0 | 0 | 2 | 2 |
| Otu59 | Unclassified | | | | | 0 | 0 | 0 | 2 |
| Otu60 | Proteobacteria | Deltaproteobacteria | Desulfuromonadales | Geobacteraceae | *Geobacter* | 0 | 1 | 0 | 0 |
| Otu61 | Actinobacteria | Actinobacteria | Rubrobacteridae |  |  | 0 | 1 | 0 | 0 |
| Otu62 | Unclassified | | | | | 0 | 0 | 0 | 1 |
| Otu63 | Bacteroidetes | Cytophagia | Cytophagales | Cytophagaceae | *Adhaeribacter* | 0 | 3 | 0 | 0 |
| Otu64 | Proteobacteria | Betaproteobacteria | Burkholderiales | Comamonadaceae | *Verminephrobacter* | 0 | 0 | 1 | 2 |
| Otu65 | Firmicutes | Bacilli | Bacillales | [Staphylococcaceae](http://en.wikipedia.org/wiki/Staphylococcaceae) | *Jeotgalicoccus* | 0 | 1 | 0 | 0 |
| Otu66 | Unclassified | | | | | 0 | 0 | 0 | 1 |
| Otu67 | Proteobacteria | Betaproteobacteria | Methylophilales | Methylophilaceae | *Methylophilus* | 0 | 0 | 7 | 9 |
| Otu68 | Unclassified | | | | | 0 | 0 | 1 | 1 |
| Otu69 | Unclassified | | | | | 0 | 0 | 1 | 1 |
| Otu70 | Proteobacteria | Deltaproteobacteria | Desulfuromonadales | Geobacteraceae | *Geobacter* | 0 | 0 | 0 | 1 |
| Otu71 | Proteobacteria | Gammaproteobacteria | Xanthomonadales | Xanthomonadaceae |  | 0 | 0 | 0 | 1 |
| Otu72 | Unclassified | | | | | 0 | 0 | 0 | 2 |
| Otu73 | Unclassified | | | | | 0 | 0 | 1 | 2 |
| Otu74 | Unclassified | | | | | 0 | 0 | 1 | 1 |
| Otu75 | Unclassified | | | | | 0 | 0 | 0 | 1 |
| Otu76 | Proteobacteria | Betaproteobacteria |  |  |  | 0 | 0 | 0 | 1 |
| Otu77 | Unclassified | | | | | 0 | 0 | 1 | 0 |
| Otu78 | Unclassified | | | | | 0 | 0 | 0 | 1 |
| Otu79 | Proteobacteria | Alphaproteobacteria | Rhizobiales | Methylobacteriaceae | *Methylobacterium* | 0 | 0 | 0 | 1 |
| Otu80 | Proteobacteria | Alphaproteobacteria | Rhizobiales | Methylobacteriaceae | *Methylobacterium* | 0 | 1 | 1 | 0 |
| Otu81 | Unclassified | | | | | 0 | 0 | 0 | 1 |
| Otu82 | Proteobacteria | Alphaproteobacteria |  |  |  | 0 | 0 | 1 | 0 |
| Otu83 | Firmicutes | Bacilli | Lactobacillales | Lactobacillaceae |  | 0 | 0 | 1 | 0 |
| Otu84 | Proteobacteria | Gammaproteobacteria | Xanthomonadales | Sinobacteraceae | *Steroidobacter* | 0 | 0 | 1 | 0 |
| Otu85 | Bacteroidetes |  |  |  |  | 0 | 0 | 1 | 0 |
| Otu86 | Unclassified | | | | | 0 | 0 | 0 | 1 |
| Otu87 | Unclassified | | | | | 0 | 0 | 0 | 1 |
| Otu88 | Unclassified | | | | | 0 | 0 | 2 | 0 |
| Otu89 | Acidobacteria |  |  |  |  | 0 | 0 | 1 | 0 |
| Otu90 | Bacteroidetes | Flavobacteriia | Flavobacteriales | Flavobacteriaceae | *Flavobacterium* | 0 | 0 | 1 | 0 |
| Otu91 | Bacteroidetes |  |  |  |  | 0 | 0 | 1 | 0 |
| Otu92 | Proteobacteria | Gammaproteobacteria | Enterobacteriales | Enterobacteriaceae | *Enterobacter* | 0 | 0 | 3 | 0 |
| Otu93 | Firmicutes |  |  |  |  | 0 | 0 | 2 | 0 |
| Otu94 | Unclassified | | | | | 0 | 0 | 1 | 4 |
| Otu95 | Actinobacteria | Actinobacteria | Actinomycetales | Streptomycetaceae | *Streptomyces* | 0 | 0 | 1 | 0 |
| Otu96 | Proteobacteria | Betaproteobacteria | Neisseriales | Neisseriaceae | *Neisseria* | 0 | 0 | 2 | 2 |
| Otu97 | Proteobacteria | Betaproteobacteria |  |  |  | 0 | 0 | 1 | 0 |
| Otu98 | Unclassified | | | | | 0 | 0 | 1 | 1 |
| Otu99 | Firmicutes | Bacilli |  |  |  | 0 | 0 | 2 | 0 |
| Otu100 | Proteobacteria | Betaproteobacteria | Burkholderiales | [UnclassifiedBurkholderiales](http://www.ncbi.nlm.nih.gov/Taxonomy/Browser/wwwtax.cgi?mode=Undef&id=119065&lvl=3&keep=1&srchmode=1&unlock) | *Ideonella* | 0 | 0 | 2 | 0 |
| Otu101 | Proteobacteria | Alphaproteobacteria | Rhizobiales | Xanthobacteraceae | *Pseudolabrys* | 0 | 0 | 1 | 0 |
| Otu102 | Proteobacteria | Betaproteobacteria | Burkholderiales | Burkholderiaceae | *Cupriavidus* | 0 | 0 | 1 | 0 |
| Otu103 | Proteobacteria | Betaproteobacteria | Burkholderiales | Comamonadaceae | *Polaromonas* | 0 | 0 | 1 | 0 |
| Otu104 | Unclassified | | | | | 0 | 0 | 1 | 0 |
| Otu105 | Proteobacteria | Betaproteobacteria | Burkholderiales | [UnclassifiedBurkholderiales](http://www.ncbi.nlm.nih.gov/Taxonomy/Browser/wwwtax.cgi?mode=Undef&id=119065&lvl=3&keep=1&srchmode=1&unlock) | *Tepidimonas* | 0 | 0 | 0 | 1 |
| Otu106 | Proteobacteria | Gammaproteobacteria | Pseudomonadales | Pseudomonadaceae | *Pseudomonas* | 1 | 0 | 0 | 0 |
| Otu107 | Proteobacteria | Alphaproteobacteria | Rhizobiales | Methylobacteriaceae | *Methylobacterium* | 2 | 0 | 0 | 0 |
| Otu108 | Proteobacteria | Gammaproteobacteria | Legionellales | Coxiellaceae | *Coxiella* | 1 | 0 | 0 | 0 |
| Otu109 | Actinobacteria | Rubrobacteridae |  |  |  | 1 | 0 | 0 | 0 |
| Otu110 | Proteobacteria | Gammaproteobacteria | Xanthomonadales | Sinobacteraceae | *Nevskia* | 2 | 0 | 0 | 0 |
| Otu111 | Acidobacteria |  |  |  |  | 1 | 0 | 0 | 0 |
| Otu112 | Unclassified | | | | | 0 | 0 | 0 | 1 |
| Otu113 | Unclassified | | | | | 0 | 0 | 0 | 1 |
| Otu114 | Actinobacteria | Actinobacteria | Actinomycetales | Microbacteriaceae | *Plantibacter* | 1 | 0 | 0 | 0 |
| Otu115 | Proteobacteria | Alphaproteobacteria | Rhodospirillales | Rhodospirillaceae | *Azospirillum* | 0 | 0 | 1 | 1 |
| Otu116 | Unclassified | | | | | 0 | 0 | 1 | 1 |
| Otu117 | Proteobacteria | Alphaproteobacteria | Caulobacterales | Caulobacteraceae | *Phenylobacterium* | 1 | 0 | 0 | 0 |
| Otu118 | Proteobacteria | Gammaproteobacteria |  |  |  | 0 | 0 | 0 | 1 |
| Otu119 | Unclassified | | | | | 0 | 0 | 1 | 1 |
| Otu120 | Unclassified | | | | | 1 | 0 | 0 | 0 |
| Otu121 | Unclassified | | | | | 1 | 0 | 0 | 0 |
| Otu122 | Proteobacteria | Gammaproteobacteria | Pseudomonadales | Pseudomonadaceae | *Pseudomonas* | 0 | 0 | 2 | 2 |
| Otu123 | Gemmatimonadetes | Gemmatimonadetes | Gemmatimonadales | Gemmatimonadaceae | *Gemmatimonas* | 1 | 0 | 0 | 0 |
| Otu124 | Proteobacteria | Gammaproteobacteria | Xanthomonadales | Xanthomonadaceae | *Luteimonas* | 1 | 0 | 0 | 0 |
| Otu125 | Proteobacteria | Alphaproteobacteria | Rhizobiales | Rhodobiaceae |  | 0 | 0 | 1 | 1 |
| Otu126 | Unclassified | | | | | 0 | 0 | 0 | 1 |
| Otu127 | Unclassified | | | | | 1 | 0 | 0 | 0 |
| Otu128 | Proteobacteria | Alphaproteobacteria | Sphingomonadales | Sphingomonadaceae | *Novosphingobium* | 0 | 0 | 0 | 1 |
| Otu129 | Actinobacteria | Actinobacteria | Actinomycetales | Intrasporangiaceae | *Terrabacter* | 0 | 0 | 1 | 1 |
| Otu130 | Unclassified | | | | | 0 | 0 | 1 | 1 |
| Otu131 | Unclassified | | | | | 0 | 0 | 0 | 1 |
| Otu132 | Proteobacteria |  |  |  |  | 0 | 0 | 1 | 1 |
| Otu133 | Acidobacteria |  |  |  |  | 0 | 0 | 0 | 2 |
| Otu134 | Actinobacteria | Actinobacteria | Actinomycetales | Dermacoccaceae | *Dermacoccus* | 0 | 0 | 0 | 1 |
| Otu135 | Firmicutes | Clostridia | Clostridiales | Lachnospiraceae |  | 0 | 0 | 0 | 1 |
| Otu136 | Actinobacteria | Actinobacteria | Actinomycetales | Dermabacteraceae | *Brachybacterium* | 1 | 0 | 0 | 0 |
| Otu137 | Proteobacteria | Gammaproteobacteria | Enterobacteriales | Enterobacteriaceae | *Escherichia* | 3 | 0 | 0 | 0 |
| Otu138 | Proteobacteria | Alphaproteobacteria | Rhizobiales | Rhizobiaceae | *Rhizobium* | 0 | 0 | 0 | 1 |
| Otu139 | Proteobacteria | Betaproteobacteria | Burkholderiales | Oxalobacteraceae |  | 0 | 0 | 2 | 1 |
| Otu140 | Proteobacteria | Deltaproteobacteria |  |  |  | 0 | 0 | 0 | 1 |
| Otu141 | Bacteroidetes | Sphingobacteriia | Sphingobacteriales |  |  | 1 | 0 | 0 | 0 |
| Otu142 | Unclassified | | | | | 2 | 0 | 0 | 0 |
| Otu143 | Actinobacteria | Rubrobacteridae | Solirubrobacterales | Solirubrobacteraceae | *Solirubrobacter* | 1 | 0 | 0 | 0 |
| Otu144 | Chloroflexi |  |  |  |  | 0 | 0 | 1 | 1 |
| Otu145 | Actinobacteria | Actinobacteria | Actinomycetales | Microbacteriaceae | *Frigoribacterium* | 1 | 0 | 0 | 0 |
| Otu146 | Unclassified | | | | | 0 | 0 | 0 | 1 |
| Otu147 | Unclassified | | | | | 1 | 0 | 0 | 0 |
| Otu148 | Proteobacteria | Betaproteobacteria |  |  |  | 0 | 0 | 0 | 1 |
| Otu149 | Unclassified | | | | | 0 | 0 | 0 | 1 |
| Otu150 | Proteobacteria | Betaproteobacteria | Burkholderiales | Comamonadaceae | *Rhodoferax* | 0 | 0 | 1 | 1 |
| Otu151 | Unclassified | | | | | 0 | 0 | 0 | 1 |
| Otu152 | Unclassified | | | | | 0 | 1 | 0 | 0 |
| Otu153 | Verrucomicrobia | UnclassifiedVerrucomicrobia |  |  |  | 0 | 0 | 0 | 1 |
| Otu154 | Firmicutes | Bacilli | Lactobacillales | Leuconostocaceae | *Leuconostoc* | 0 | 0 | 0 | 1 |
| Otu155 | Unclassified | | | | | 0 | 1 | 0 | 0 |
| Otu156 | Proteobacteria | Gammaproteobacteria | Xanthomonadales | Xanthomonadaceae | *Arenimonas* | 0 | 1 | 0 | 0 |
| Otu157 | Proteobacteria | Betaproteobacteria | Burkholderiales | Mitsuaria |  | 0 | 2 | 0 | 0 |
| Otu158 | Unclassified | | | | | 0 | 1 | 0 | 0 |
| Otu159 | Actinobacteria | Actinobacteria | Actinomycetales | Microbacteriaceae | *Microbacterium* | 0 | 1 | 0 | 0 |
| Otu160 | Bacteroidetes | Sphingobacteriia |  |  |  | 0 | 1 | 0 | 0 |
| Otu161 | Chloroflexi |  |  |  |  | 0 | 1 | 0 | 0 |
| Otu162 | Cyanobacteria |  |  |  |  | 0 | 1 | 0 | 0 |
| Otu163 | Unclassified | | | | | 0 | 0 | 0 | 1 |
| Otu164 | Proteobacteria | Alphaproteobacteria | Rhizobiales | Rhizobiaceae | *Shinella* | 0 | 1 | 0 | 0 |
| Otu165 | Proteobacteria | Alphaproteobacteria | Caulobacterales | Caulobacteraceae | *Asticcacaulis* | 0 | 1 | 0 | 0 |
| Otu166 | Firmicutes | Clostridia | Clostridiales | Eubacteriaceae | *Eubacterium* | 0 | 1 | 0 | 0 |
| Otu167 | Actinobacteria | Actinobacteria | Actinomycetales | Micrococcaceae | *Kocuria* | 0 | 0 | 0 | 2 |
| Otu168 | Bacteroidetes | Sphingobacteriia |  |  |  | 0 | 0 | 1 | 2 |
| Otu169 | Unclassified | | | | | 0 | 1 | 0 | 0 |
| Otu170 | Unclassified | | | | | 0 | 0 | 0 | 1 |
| Otu171 | Proteobacteria | Betaproteobacteria | Neisseriales | Neisseriaceae | *Prolinoborus* |  | 1 | 0 | 0 |
| Otu172 | Actinobacteria | Actinobacteria | Actinomycetales | Propionibacterineae | *Aeromicrobium* | 0 | 0 | 1 | 1 |
| Otu173 | Unclassified | | | | | 0 | 0 | 1 | 1 |
| Otu174 | Firmicutes | Bacilli | Bacillales | Paenibacillaceae | *Brevibacillus* | 0 | 0 | 0 | 1 |
| Otu175 | Unclassified | | | | | 0 | 0 | 0 | 1 |
| Otu176 | Unclassified | | | | | 0 | 0 | 0 | 1 |
| Otu177 | Bacteroidetes | Flavobacteriia | Flavobacteriales | Flavobacteriaceae | *Chryseobacterium* | 0 | 0 | 0 | 2 |
| Otu178 | Bacteroidetes |  |  |  |  | 0 | 1 | 0 | 0 |
| Otu179 | Unclassified | | | | | 0 | 1 | 0 | 0 |
| Otu180 | Unclassified | | | | | 0 | 1 | 0 | 0 |
| Otu181 | Actinobacteria | Actinobacteria | Actinomycetales | Micrococcaceae | *Micrococcus* | 0 | 0 | 2 | 2 |
| Otu182 | Proteobacteria | Alphaproteobacteria | Rhizobiales | Phyllobacteriaceae | *Phyllobacterium* | 0 | 0 | 0 | 1 |
| Otu183 | Deinococcus-Thermus | Deinococci | Thermales | Thermaceae | *Meiothermus* | 0 | 3 | 0 | 0 |
| Otu184 | Proteobacteria | Alphaproteobacteria | Sphingomonadales | Sphingomonadaceae | *Sphingomonas* | 0 | 0 | 0 | 3 |
| Otu185 | Unclassified | | | | | 0 | 0 | 1 | 1 |
| Otu186 | Unclassified | | | | | 0 | 0 | 0 | 1 |
| Otu187 | Unclassified | | | | | 0 | 1 | 0 | 0 |
| Otu188 | Unclassified | | | | | 0 | 1 | 0 | 0 |
