# Supplementary material for: Maize Endophytic Bacterial Diversity as Affected by Soil Cultivation History
Source: Front Microbiol. 2018 Mar 16;9:484. doi: 10.3389/fmicb.2018.00484 (PMC5890191; doi:10.3389/fmicb.2018.00484)
Supplement: Supplementary file 3 [file Table3.docx]

|  | Plant growth promoting characteristic | | | | | |
| --- | --- | --- | --- | --- | --- | --- |
| Genus | Nitrogen Fixation | P solubilization | Siderophore production | IAA production | ACC deaminase activity | Biocontrol activity |
| *Sphingomonas* | +  (Yang et al. 2014) | +  (Midekssa et al. 2015) | +  (Sukweenadhi et al. 2015) | +  (Asaf et al. 2017) | +  (Khan et al. 2016) | +  (Khan et al. 2017) |
| *Burkholderia* | +  (Talbi et al. 2013) | +  (Acevedo et al. 2014) | +  (Esmaeel et al. 2016) | +  (Jiang et al. 2008) | +  (Palaniappan et al. 2010) | +  (Bulgari et al. 2011) |
| *Candidatus*  Glomeribacter | +  (Dedysh et al. 2004) | - | - | - | - | +  (Dames and Ridsdale, 2012) |
| *Dyella* | - | - | - | +  (Contreras et al. 2016) | +  (Palaniappan et al. 2010) | - |
| *Herbaspirillum* | +  (Dedysh et al. 2004) | +  (Estrada et al. 2013) | +  (Rosconi et al. 2013) | +  (Zhan et al. 2016) | +  (Singh et al. 2015) | +  (Brusamarello-Santos et al. 2012) |
| *Bradyrhizobium* | +  (Bedmar et al. 2005) | +  (Ahemad and Kibret 2014) | +  (Ahemad and Kibret 2014) | +  (Seneviratne et al. 2016) | +  (Nascimento et al. 2016) | - |
| *Staphylococcus* | - | +  (Acevedo et al. 2014) | +  (Tripathi et al. 2014) | +  (Srivastava et al. 2013) | +  (Srivastava et al. 2013) | - |
| *Methylophilus* | - | +  (Agafonova et al. 2013) | +  (Madhaiyan et al. 2009) | +  (Madhaiyan et al. 2009) | +  (Madhaiyan et al. 2013) | +  (Agafonova et al. 2016) |
| *Variovorax* | - | +  (Nadeem et al. 2016) | +  (Kurth et al. 2016) | +  (Jiang et al. 2012) | +  (Singh et al. 2015) | +  (Han et al. 2011) |
| *Streptococcus* | - | +  (Wahid et al. 2016) | +  (Retamales et al. 2012) | - | - | +  (Wani et al. 2015) |
| *Bacillus* | +  (Sivasakthi et al. 2014) | +  (Sivasakthi et al. 2014) | +  (Yu et al. 2017) | +  (Ahmad et al. 2008) | +  (Khan et al. 2016) | +  (Sivasakthi et al. 2014) |
| *Chitinophaga* | - | +  (Chung et al. 2012) | +  (Sauvêtre and Schröder 2015). | +  (Sauvêtre and Schröder 2015) | - | - |

Table S3. Plant growth promoting activities described for the 12 main genera found in roots of maize plants grown in fallow and maize-cultivated soils.

References for Table 2S

Acevedo, E., Galindo-Castaneda, T., Prada, F., Navia, M., and Romero, H. M. (2014). Phosphate-solubilizing microorganisms associated with the rhizosphere of oil palm (*Elaeis guineensis* Jacq.) in Colombia. *Appl. Soil Ecol.* 80, 26-33.

Agafonova, N. V., Kaparullina, E. N., Doronina, N. V., and Trotsenko, Y. A. (2013). Phosphate-solubilizing activity of aerobic methylobacteria. *Microbiology* 82, 864-867.

Agafonova, N. V., Doronina, N. V., and Trotsenko, Y. A. (2016). Enhanced resistance of pea plants to oxidative stress caused by paraquat during colonization by aerobic methylobacteria. ***Appl. Biochem. Microbiol.*** 52, 199-204

Ahemad, M., and Kibret, M. (2014). Mechanisms and applications of plant growth promoting rhizobacteria: current perspective. *JKSUES.* 26, 1-20.

Ahmad, F., Ahmad, I., and Khan, M. S. (2008). Screening of free-living rhizospheric bacteria for their multiple plant growth promoting activities. *Microbiol. Res.* 136, 173-181.

Asaf, S., Khan, M. A., Khan, A. L., Waqas, M., Shahzad, R., Kim, A. Y., et al. (2017). Bacterial endophytes from arid land plants regulate endogenous hormone content and promote growth in crop plants: an example of *Sphingomonas* sp. and *Serratia marcescens*. ***J. Plant Interact.*** 12, 31-38.

Bedmar, E. J., Robles, E. F., and Delgado, M. J. (2005). The complete denitrification pathway of the symbiotic, nitrogen-fixing bacterium *Bradyrhizobium japonicum*. ***Biochem. Soc. Trans*. 33,** 141-144.

Brusamarello-Santos, L. C. C., Pacheco, F., Aljanabi, S. M. M., Monteiro, R. A., Cruz, L. M., Baura, V. A., et al. (2012). Differential gene expression of rice roots inoculated with the diazotroph *Herbaspirillum seropedicae*. *Plant Soil* 356, 113-125.

Bulgari, D., Casati, P., Crepaldi, P., Daffonchio, D., Quaglino, F., Brusetti, L., and Bianco, P. A. (2011). Restructuring of endophytic bacterial communities in grapevine yellows-diseased and recovered *Vitis vinifera* L. plants. *Appl. Environ. Microbiol.* 77, 5018-5022.

Chung, E. J., Park, T. S., Jeon, C. O., and Chung, Y. R. (2012). *Chitinophaga oryziterrae* sp. nov., isolated from the rhizosphere soil of rice (*Oryza sativa* L.). *Int. J. Syst. Evol. Microbiol*. 62, 3030-3035.

Contreras, M., Loeza, P. D., Villegas, J., Farias, R., and Santoyo, G. (2016). A glimpse of the endophytic bacterial diversity in roots of blackberry plants (*Rubus fruticosus*). *Genet. Mol. Res.* 15, ID15038542.

Dames, J. F., and Ridsdale, C. J. (2012). What we know about arbuscular mycorhizal fungi and associated soil bacteria. *Afr. J. Biotechnol.* 11, 13753-13760.

Dedysh, S. N., Ricke, P., and Liesack, W. (2004). NifH and NifD phylogenies: an evolutionary basis for understanding nitrogen fixation capabilities of methanotrophic bacteria. *Microbiology* 150, 1301-1313.

Esmaeel, Q., Pupin, M., Kieu, N. P., Chataigné, G., Béchet, M., Deravel, J., et al. (2016). *Burkholderia* genome mining for nonribosomal peptide synthetases reveals a great potential for novel siderophores and lipopeptides synthesis. *Microbiologyopen*. 5, 512-526.

Estrada, G. A., Baldani, V. L. D., de Oliveira, D. M., Urquiaga, S., and Baldani, J. I. (2013). Selection of phosphate-solubilizing diazotrophic *Herbaspirillum* and *Burkholderia* strains and their effect on rice crop yield and nutrient uptake. *Plant Soil* 369, 115-129.

Han, J. I., Choi, H. K., Lee, S. W., Orwin, P. M., Kim, J., LaRoe, S. L., et al. (2011). Complete genome sequence of the metabolically versatile plant growth-promoting endophyte *Variovorax paradoxus* S110. *J. Bacteriol.* 193, 1183-1190.

Jiang, C. Y., Sheng, X. F., Qian, M., and Wang, Q. Y. (2008). Isolation and characterization of a heavy metal-resistant *Burkholderia* sp. from heavy metal-contaminated paddy field soil and its potential in promoting plant growth and heavy metal accumulation in metal-polluted soil. *Chemosphere* 72, 157-164.

Jiang, F., Chen, L., Belimov, A. A., Shaposhnikov, A. I., Gong, F., Meng, X., et al. (2012). Multiple impacts of the plant growth-promoting rhizobacterium *Variovorax paradoxus* 5C-2 on nutrient and ABA relations of *Pisum sativum*. *J. Exp. Bot.* 63, 6421-6430.

Khan, A. L., Halo, B. A., Elyassi, A., Ali, S., Al-Hosni, K., Hussain, J., et al. (2016). Indole acetic acid and ACC deaminase from endophytic bacteria improves the growth of *Solanum lycopersicum. Electron. J. Biotechnol*. 21, 58-64.

Khan, A. L., Waqas, M., Asaf, S., Kamran, M., Shahzad, R., Bilal, S., et al. (2017). Plant growth-promoting endophyte *Sphingomonas* sp. LK11 alleviates salinity stress in *Solanum pimpinellifolium*. ***Environ. Exp. Bot.*** 133, 58-69.

Kurth, C., Schieferdecker, S., Athanasopoulou, K., Seccareccia, I., and Nett, M. (2016). Variochelins, lipopeptide siderophores from *Variovorax boronicumulans* discovered by genome mining. *J. Nat. Prod.* 79, 865-872.

Madhaiyan, M., Poonguzhali, S., Kwon, S. W., and Sa, T. M. (2009). *Methylophilus rhizosphaerae* sp. nov., a restricted facultative methylotroph isolated from rice rhizosphere soil. *Int. J. Syst. Evol. Microbiol.* 59, 2904-2908.

Madhaiyan, M., Poonguzhali, S., Senthilkumar, M., Pragatheswari, D., Lee, K. C., and Lee, J. S. (2013). *Methylobacillus rhizosphaerae* sp. nov., a novel plant-associated methylotrophic bacterium isolated from rhizosphere of red pepper. *Antonie van Leeuwenhoek* 103, 475-484.

Midekssa, M. J., Loscher, C. R., Schmitz, R. A., and Assefa, F. (2015). Characterization of phosphate solubilizing rhizobacteria isolated from lentil growing areas of Ethiopia. *Afr. J. Agric. Res*. 9, 1637-1648.

Nadeem, S. M., Ahmad, M., Naveed, M., Imran, M., Zahir, Z. A., and Crowley, D. E. (2016). Relationship between in vitro characterization and comparative efficacy of plant growth-promoting rhizobacteria for improving cucumber salt tolerance. *Arch. Microbiol*. 198, 379-387.

Nascimento, F. X., Brígido, C., Glick, B. R., and Rossi, M. J. (2016). The role of rhizobial ACC deaminase in the nodulation process of leguminous plants. *IJAAR.* 2016, ID1369472.

Palaniappan, P., Chauhan, P. S., Saravanan, V. S., Anandham, R., and Sa, T. (2010). Isolation and characterization of plant growth promoting endophytic bacterial isolates from root nodule of *Lespedeza* sp. *Biol. Fertil. Soils*. 46, 807-816.

Retamales, J., González-Contreras, A., Salazar, S., Toranzo, A. E., and Avendaño-Herrera, R. (2012). Iron utilization and siderophore production by *Streptococcus phocae* isolated from diseased Atlantic salmon (*Salmo salar*). *Aquaculture* 364, 305-311.

Rosconi, F., Davyt, D., Martínez, V., Martínez, M., Abin‐Carriquiry, J. A., Zane, H., et al. (2013). Identification and structural characterization of serobactins, a suite of lipopeptide siderophores produced by the grass endophyte *Herbaspirillum seropedicae*. *Environ. Microbiol.* 15, 916-927.

Sauvêtre, A., and Schröder, P. (2015). Uptake of carbamazepine by rhizomes and endophytic bacteria of *Phragmites australis*. *Front. Plant Sci*. 6, 83.

Seneviratne, M., Gunaratne, S., Bandara, T., Weerasundara, L., Rajakaruna, N., Seneviratne, G., and Vithanage, M. (2016). Plant growth promotion by *Bradyrhizobium japonicum* under heavy metal stress. *S. African J. Bot*. 105, 19-24.

Singh, R. P., Shelke, G. M., Kumar, A., and Jha, P. N. (2015). Biochemistry and genetics of ACC deaminase: a weapon to “stress ethylene” produced in plants. *Front. Microbiol*. 6, 937.

Sivasakthi, S., Usharani, G., and Saranraj, P. (2014). Biocontrol potentiality of plant growth promoting bacteria (PGPR)-*Pseudomonas fluorescens* and *Bacillus subtilis*: A review*. Afr. J. Agric. Res*. 9, 1265-1277.

Srivastava, S., Verma, P. C., Chaudhry, V., Singh, N., Abhilash, P. C., Kumar, K. V., et al. (2013). Influence of inoculation of arsenic-resistant *Staphylococcus arlettae* on growth and arsenic uptake in *Brassica juncea* (L.) Czern. Var. R-46. *J. Hazard. Mater.* 262, 1039-1047.

Sukweenadhi, J., Kim, Y. J., Kang, C. H., Farh, M. E. A., Nguyen, N. L., Hoang, V. A., et al. (2015). *Sphingomonas panaciterrae* sp. nov., a plant growth-promoting bacterium isolated from soil of a ginseng field. *Arch. Microbiol*. 197, 973-981.

Talbi, C., Argandoña, M., Salvador, M., Alché, J. D., Vargas, C., Bedmar, E. J., and Delgado, M. J. (2013). *Burkholderia phymatum* improves salt tolerance of symbiotic nitrogen fixation in *Phaseolus vulgaris*. *Plant Soil* 36, 673-685.

Tripathi, A., Schofield, M. M., Chlipala, G. E., Schultz, P. J., Yim, I., Newmister, S. A., et al. (2014). Baulamycins A and B, broad-spectrum antibiotics identified as inhibitors of siderophore biosynthesis in *Staphylococcus aureus* and *Bacillus anthracis*. *J. Am. Chem. Soc*. 136, 1579-1586.

Wahid, F., Sharif, M., Khan, M. A., and Khan, M. J. (2016). Status and phosphorus solubilization potential of bacteria and arbuscular mycorrhizal fungi isolated from various locations of Khyber Pakhtunkhwa province Pak. *J. Bot*. 48, 2121-2130.

Wani, P. A., Zainab, I. O., Wasiu, I. A., and Jamiu, K. O. (2015). Chromium (VI) reduction by *Streptococcus* species isolated from the industrial area of Abeokuta, Ogun State, Nigeria. *Res. J. Microbiol*. 10, 66-75

Yang, S., Zhang, X., Cao, Z., Zhao, K., Wang, S., Chen, M., and Hu, X. (2014). Growth‐promoting *Sphingomonas paucimobilis* ZJSH1 associated with *Dendrobium officinale* through phytohormone production and nitrogen fixation. ***Microb. Biotechnol*.** 7, 611-620.

Yu, S., Teng, C., Bai, X., Liang, J., Song, T., Dong, L., et al. (2017). The optimization of siderophore production by *Bacillus* sp. PZ-1 and its potential enhancement on phytoextration of Pb from soil. [*J. Microbiol. Biotechnol.*](https://www.ncbi.nlm.nih.gov/pubmed/28633518) 27, 1500-1512.

Zhan, G., Cheng, W., Liu, W., Li, Y., Ding, K., Rao, H., et al. (2016). Infection, colonization and growth-promoting effects of tea plant (*Camellia sinensis* L.) by the endophytic bacterium *Herbaspirillum* sp. WT00C. *Afr. J. Agric. Res*. 11, 130-138.
