# Supplementary figures and images for: Maize Endophytic Bacterial Diversity as Affected by Soil Cultivation History
Source: Front Microbiol. 2018 Mar 16;9:484. doi: 10.3389/fmicb.2018.00484 (PMC5890191; doi:10.3389/fmicb.2018.00484)

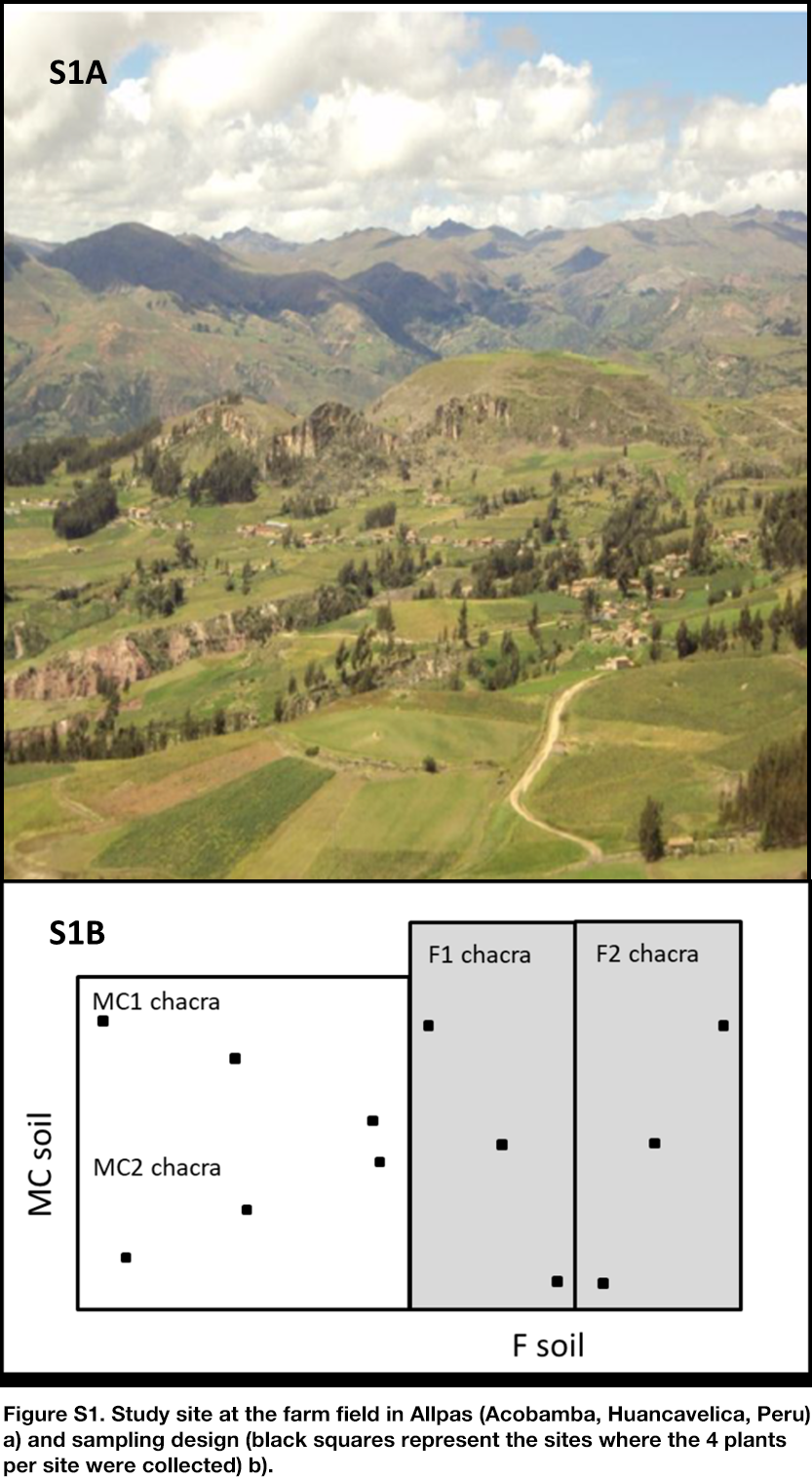

Supplement: Supplementary file 4 [file Image1.tif]
